# Xanthophyll-Rich Extract of *Phaeodactylum tricornutum* Bohlin as New Photoprotective Cosmeceutical Agent: Safety and Efficacy Assessment on In Vitro Reconstructed Human Epidermis Model

**DOI:** 10.3390/molecules28104190

**Published:** 2023-05-19

**Authors:** Antonella Smeriglio, Joseph Lionti, Mariarosaria Ingegneri, Bruno Burlando, Laura Cornara, Federica Grillo, Luca Mastracci, Domenico Trombetta

**Affiliations:** 1 Department of Chemical, Biological, Pharmaceutical and Environmental Sciences (ChiBioFarAm), University of Messina, Viale Ferdinando Stagno d’Alcontres 31, 98166 Messina, Italy; antonella.smeriglio@unime.it (A.S.); mariarosaria.ingegneri@unime.it (M.I.); domenico.trombetta@unime.it (D.T.); 2 Archimede Ricerche Srl, Corso Italia 220, 18033 Camporosso, Italy; j.lionti@archimedericerche.com; 3 Department of Experimental Medicine (DIMES), University of Genova, Via Leon Battista Alberti, 2, 16132 Genova, Italy; 4 Department of Pharmacy—DIFAR, University of Genova, Viale Benedetto XV 3, 16132 Genova, Italy; 5 Department of Earth, Environment and Life Sciences (DISTAV), University of Genova, Corso Europa 26, 16132 Genova, Italy; 6 Pathology Unit, Department of Surgical Sciences and Integrated Diagnostics (DISC), University of Genova, 16132 Genova, Italy; federica.grillo@unige.it (F.G.); luca.mastracci@unige.it (L.M.); 7 IRCCS Ospedale Policlinico San Martino, 16132 Genova, Italy

**Keywords:** microalgae, *Phaeodactylum tricornutum* bohlin, microscopy, phytochemical analysis, carotenoids, fucoxanthin, antioxidant activity, photostability, phototoxicity, photoprotection

## Abstract

The nutritional and health properties of algae make them perfect functional ingredients for nutraceutical and cosmeceutical applications. In this study, the *Phaeodactylum tricornutum* Bohlin (Phaeodactylaceae), a pleiomorphic diatom commonly found in marine ecosystems, was investigated. The in vitro culture conditions used favoured the fusiform morphotype, characterized by a high accumulation of neutral lipids, as detected by fluorescence microscopy after BODIPY staining. These data were confirmed by HPLC-DAD-APCI-MS/MS analyses carried out on the ethanolic extract (PTE), which showed a high content of xanthophylls (98.99%), and in particular of fucoxanthin (Fx, 6.67 g/100 g PTE). The antioxidant activity (ORAC, FRAP, TEAC and β-carotene bleaching) and photostability of PTE and Fx against UVA and UVB rays were firstly evaluated by in vitro cell-free assays. After this, phototoxicity and photoprotective studies were carried out on in vitro reconstructed human epidermidis models. Results demonstrated that PTE (0.1% Fx) and 0.1% Fx, both photostable, significantly (*p* < 0.05) reduce oxidative and inflammatory stress markers (ROS, NO and IL-1α), as well as cytotoxicity and sunburn cells induced by UVA and UVB doses simulating the solar radiation, with an excellent safety profile. However, PTE proved to be more effective than Fx, suggesting its effective and safe use in broad-spectrum sunscreens.

## 1. Introduction

The nutritional and health properties of algae make them perfect functional ingredients for nutraceutical and cosmeceutical applications. They have a high energy value and are rich source of nutritional and biologically active substances such as proteins, fats, carbohydrates, vitamins, macro- and microelements as well as chlorophylls and carotenoids [1]. Recently, there is an ever-increasing interest in the healthful use of algae extracts, in particular for their richness in secondary metabolites, and this has led to focus the attention on microscopic algae that can be cultivated in bioreactors [2].

*Phaeodactylum tricornutum* Bohlin (Phaeodactylaceae) is one of the most studied marine diatoms due to its high biomass productivity allowing large scale growth in bioreactors, and the possibility of modulating the algal biochemical composition by manipulating culture conditions [3]. *P. tricornutum* (PT) is a pennate diatom commonly found in marine ecosystems, where it exists in three different morphotypes, namely oval, fusiform, and triradiate, depending on environmental conditions [4]. It has a remarkable nutritional value for its content in protein (30–70%), sugars (10–30%), and fat (20–30%), and is an important source of the omega-3 eicosapentaenoic acid, the carotenoid fucoxanthin (Fx), and the polysaccharide chrysolaminarin [5].

Different studies have been dedicated to PT’s biochemical composition and biological properties, especially for nutritional and skin care purposes [6]. In a preclinical study on mice, a diet enriched with the PT’s main secondary metabolites has induced positive effects on the microbiota and the short-chain fatty acid composition [5]. Fx extracted from PT has been found to exert in vitro antiproliferative and antioxidant effects suggesting that the microalgae extract could be used as a nutraceutical in human nutrition [7].

The dermatologic and cosmetic field is another healthcare and commercially relevant context within which PT has been studied. The hydrolysed extract has promoted in vitro anti-inflammatory and antioxidant effects on human dermal fibroblasts, indicating its possible use in skin care products [8]. In another study, a Fx concentrate derived from PT has shown antiaging potentials in vitro, by increasing procollagen synthesis and lowering matrix metalloproteinase expression, and accordingly, has reduced wrinkles, and improved skin moisture and elasticity, when tested on women [9]. In addition, Fx has been proved to induce in vitro and in vivo anti-skin pigmentation activity by inhibiting the melanin biosynthesis pathway [10].

However, to date no studies are available about the photoprotective role of PT. Considering this, we decided to investigate this new cosmeceutical application. To this aim, we studied the chemical composition and biological properties of a xanthophyll-rich, ethanolic extract (PTE) obtained from the fusiformis morphotype of the diatom cultivated in a bioreactor. The diatom morphotype was analysed by light (LM) and scanning electron microscopy (SEM). The storage of neutral lipids was evaluated by fluorescence microscopy. PTE was chemically characterized by high pressure liquid chromatography with diode array detection coupled to atmospheric pressure chemical ionization tandem-mass spectrometry (HPLC-DAD-APCI-MS/MS) and titrated in Fx, the most abundant compound. PTE and Fx were tested for in vitro antioxidant power and photostability by assays based on different environments and reaction mechanisms. Finally, safety and efficacy as skin photoprotective agents against UVA and UVB radiations, simulating the solar radiance exposition, were assessed on reconstructed human epidermidis models.

## 2. Results

### 2.1. Micromorphological Analysis of P. tricornutum

In the cultures of PT, the triradiate morphotype that is usually observed during the cultivation phase was gradually substituted by the fusiformis morphotype due to the self-shading of the growing culture, lowering the amount of available light, and therefore reducing growing rate in favour of a higher Fx production [11,12]. The fusiformis shape is the commonest in large PT production plants that use natural light, since succession of light and shading prevents the development of the triradiate form, which is a symptom of a highly illuminated culture, or of the ovoidal form, which instead manifest a stress condition [13]. The carotenoid production in the fusiformis morphotype is a key aspect for favouring this shape during large scale cultivation over the other two counterparts. Following the exponential phase, fusiformis cells can activate a metabolic switch caused by self-shading. This triggers the accumulation of light harvesting pigments, such as carotenoids, notably Fx [14,15].

**Figure 1:**
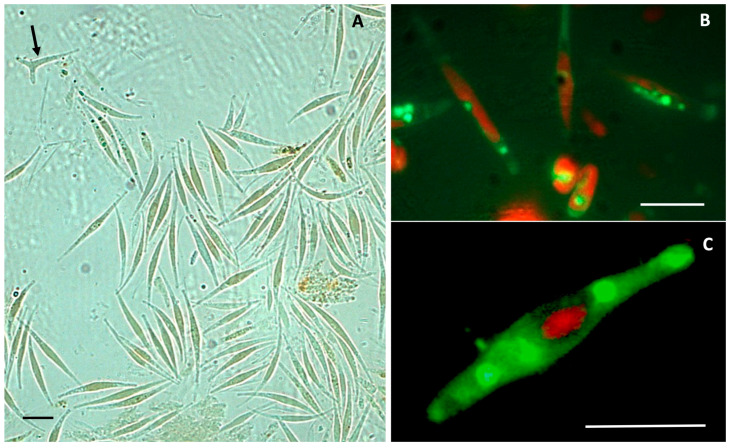
Light micrographs of *P. tricornutum* from a freeze-dried sample diluted in water for observation. (**A**) Many fusiformis cells dominate the field of observation, while only one isolated triradiate form is visible (dark arrow). (**B**,**C**) Magnified fluorescence images of *P. tricornutum* cells, showing autofluorescent chlorophyll in red, and neutral lipid storage in green (BODIPY fluorescent stain). Bar = 10 µm.

**Figure 2:**
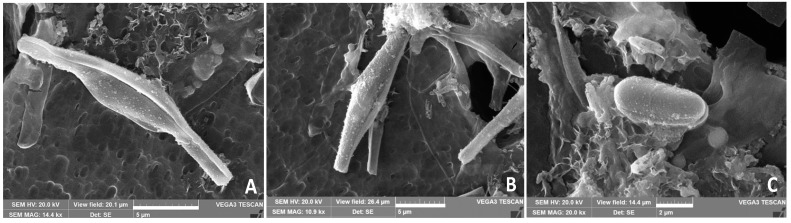
Scanning electron micrographs of a *P. tricornutum* cell culture. (**A**,**B**) Cells of the fusiformis morphotype in the process of cytodieresis. (**C**) Detail of a cell of the ovoidal morphotype. (**A**,**B**) Scale bar = 5 µm; (**C**) Scale bar = 2 µm.

The predominant fusiformis morphotype is well documented in the light microscope observation of a freeze-dried sample of biomass, in which 99% of cells were displaying this shape (Figure 1A), while only one triradiate cell was visible (Figure 1A, arrow). The fusiformis morphotype showed an increased accumulation of neutral lipids, stained in bright green by BODIPY, and the autofluorescent chlorophyll in red (Figure 1B,C). The freeze-dried biomass was later used for carotenoid extraction. In SEM micrographs, the fusiform morphotype was frequently found under division (Figure 2A,B), while the doubling of the ovoidal form was only rarely observed (Figure 2C).

### 2.2. Phytochemical Analysis

Given the conspicuous presence of light harvesting pigments detected by microscopic analyses, the phytochemical analysis focused on the identification of these secondary metabolites, which appears to be the most expressed in *P. tricornutum*. The phytochemical profile of the PT ethanolic extract (PTE), elucidated by HPLC-DAD-APCI-MS/MS analysis, is shown in Table 1.

Comparing the retention times, UV-Vis and mass spectra of analytes with those of commercially available standards (where available), as well as with the literature data and UV–Vis and mass spectra databases, nine compounds were tentatively identified.

A semi-quantitative analysis, expressing the results as mean peak area percentage (%) of each compound, with respect to the total area of identified compounds based on diode array detection chromatograms acquired at 450 nm, was carried out (Table 1, see also Figure S1 in Supplementary). Three of them, chlorophyllide a, chlorophyll c1, and chlorophyll a, belonging to the green pigments class of chlorophylls, were poorly represented in this algal morphotype, able to shift its metabolism in favour of the carotenoids’ production (0.80% chlorophylls vs. 99.20% carotenoids, Table 1) based on the particular growth cultivation conditions it required [11,12]. Regarding carotenoids, PTE results in a rich source of xantophylls and in particular of Fx. (E)-Fx was the most abundant detected compound (77.45%), followed by its two cis isomers, 9Z-Fx (15.44%) and 13Z-Fx (5.18%). Finally, other two minor xanthophylls, (E)-diadinoxanthin (0.47%) and (E)-diatoxanthin (0.45%), as well as β-carotene (0.22%), the only compound belonging to the sub-class of the carotenes, were detected.

Given the conspicuous presence of Fx and the commercial availability of the reference standard, a titration of the PTE to quantify the most abundant bioactive compound, was carried out. The extract under examination proved to be a very rich source of this carotenoid, containing 6.61 g of Fx/100 g PTE.

### 2.3. Biological Activity

Given the conspicuous presence of Fx in the extract under examination, the biological activity of PTE was investigated by comparing the activity of the plant-complex to that of pure Fx, in order to verify whether the biological activity was totally ascribed or less to this carotenoid. At this purpose, Fx was always tested at the same concentration in which it is present within PTE.

#### 2.3.1. In Vitro Cell-Free Antioxidant Activity

**Figure 3:**
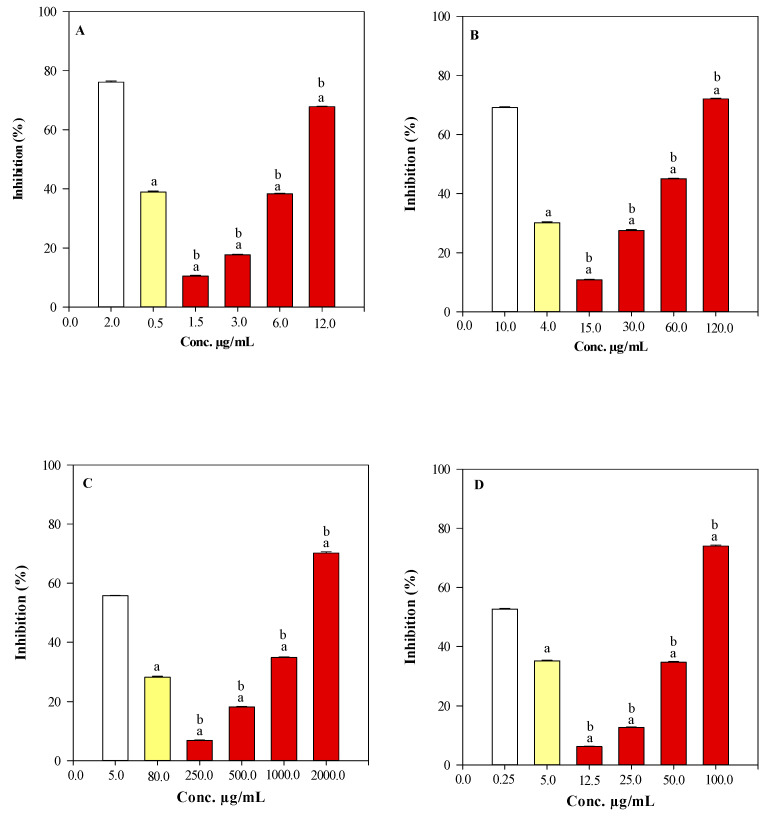
Antioxidant and free radical-scavenging activity of *Phaeodactylum tricornutum* ethanolic extract (PTE, red bars) and fucoxanthin (Fx, yellow bars) in comparison with the reference compound (white bars), that is the trolox for ORAC (**A**), TEAC (**B**), and FRAP (**C**) assays, and butylhydroxytoluene (BHT) for BCB assay (**D**). Three independent experiments in triplicate (*n* = 3) were carried out for each tested sample. ^a^ *p* < 0.05 vs. trolox or BHT; ^b^ *p* < 0.05 vs. Fx.

The antioxidant activity of PTE and Fx was investigated by four in vitro cell-free assays based on different mechanisms and reaction environments (Figure 3).

In particular, the oxygen radical absorbance capacity (ORAC, Figure 3A), the trolox equivalent antioxidant capacity (TEAC, Figure 3B), the ferric-reducing antioxidant power (FRAP, Figure 3C) and the ability of PTE and Fx to counteract the β-carotene bleaching (BCB, Figure 3D), were investigated.

A preliminary antioxidant screening was carried out only on PTE in order to find the most appropriate concentration range that would allow calculating the half-inhibitory concentration of the oxidant/radical activity (IC_50_) with the respective 95% confidence limits (C.L.). Subsequently, Fx was tested, in the same conditions, at the same concentrations present within the PTE chosen concentration range.

Figure 1 shows the results of the four antioxidant tests, comparing the four tested concentrations of the extract under examination with respect to the reference standard (white bar) trolox (Figure 3A–C) and BHT (Figure 3D), and Fx (yellow bar). Reference standards and Fx are shown in the graph bars only at one concentration, specifically the highest for the reference standards, and the closest to the IC_50_ of PTE for Fx, to make a more immediate comparison with the plant-complex activity.

In all the tests carried out, PTE showed a strong antioxidant activity, highlighting a concentration-dependent behaviour. Results were statistically significant (*p* < 0.05) both with respect to the reference standards and Fx in all assays carried out (Figure 3A–D). Interestingly Fx, in all the tests carried out, despite being tested at the concentration corresponding to that present within PTE, which showed 50% of the antioxidant activity (IC_50_), showed always a clearly lower activity, ranging between 28.45% and 38.87% inhibition. This indirectly demonstrated that, although Fx is the predominant compound of the extract, it does not contribute exclusively to the biological activity of the plant-complex. The strongest activity of PTE was found in the ORAC assay (IC_50_ 8.36 µg/mL, C.L. 6.71–10.42), followed by TEAC (IC_50_ 62.6 µg/mL, C.L. 51.86–75.57), BCB (IC_50_ 79.87 µg/mL, C.L. 67.43–100.25) and FRAP (IC_50_ 1218.35 µg/mL, C.L. 1033.33–1440.69) assay, showing a greater propensity of PTE, and therefore of its constituents, to exert greater activity by antioxidant mechanisms based on hydrogen atoms transfer (ORAC and BCB) or on hydrogen atoms and electrons transfer (TEAC, Figure 3B). Moreover, PTE showed a strong anti-peroxidase activity effectively inhibiting the heat-induced peroxidation of linoleic acid in the BCB assay (Figure 3D).

Based on the results of this in vitro antioxidant screening, which showed the maximum IC_50_ of the PTE to be about 1 mg/mL, we selected 0.1% Fx as the suitable concentration to be tested in the subsequent experiments, which among other things, falls perfectly within the range of concentrations (0.01–1%) generally used for antioxidants in cosmetic formulations [16].

#### 2.3.2. Photostability Studies

**Figure 4:**
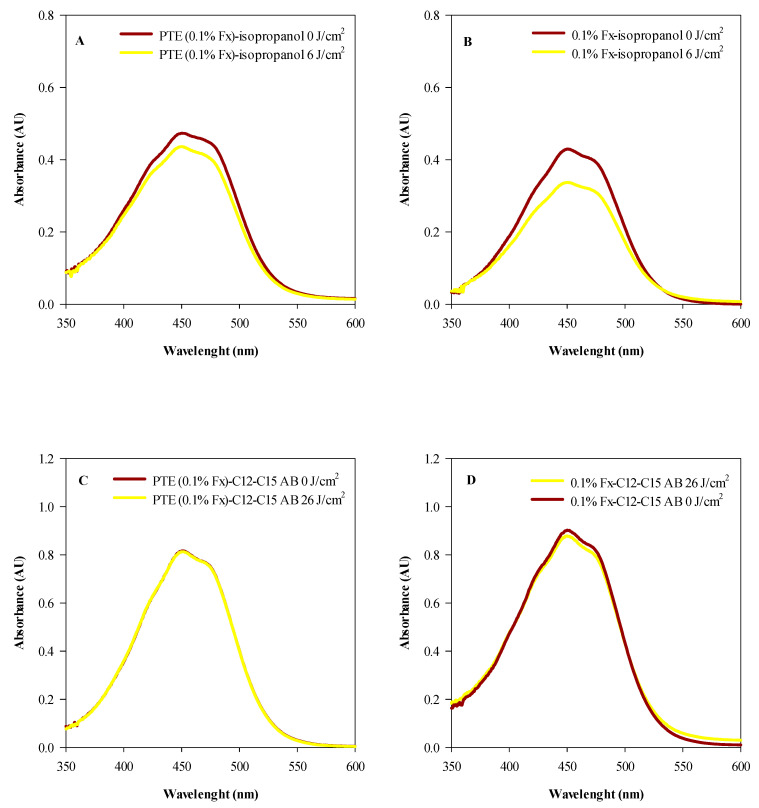
Photostability studies based on the electromagnetic spectra of tested samples irradiated (yellow line) or not (red line) with 6 J/cm^2^ (**A**,**B**) and 26 J/cm^2^ UVA rays (**C**,**D**). (**A**) Isopropanol solution of *Phaeodactylum tricornutum* ethanolic extract (PTE, 0.1% Fx); (**B**) Isopropanol solution of 0.1% fucoxanthin (Fx); (**C**) PTE (0.1% Fx) dissolved in C12–C15 alkylbenzoate (C12–C15 AB); (**D**) 0.1% Fx dissolved in C12–C15 alkylbenzoate (C12–C15 AB). Three independent experiments in triplicate (*n* = 3) were carried out for each tested sample.

**Figure 5:**
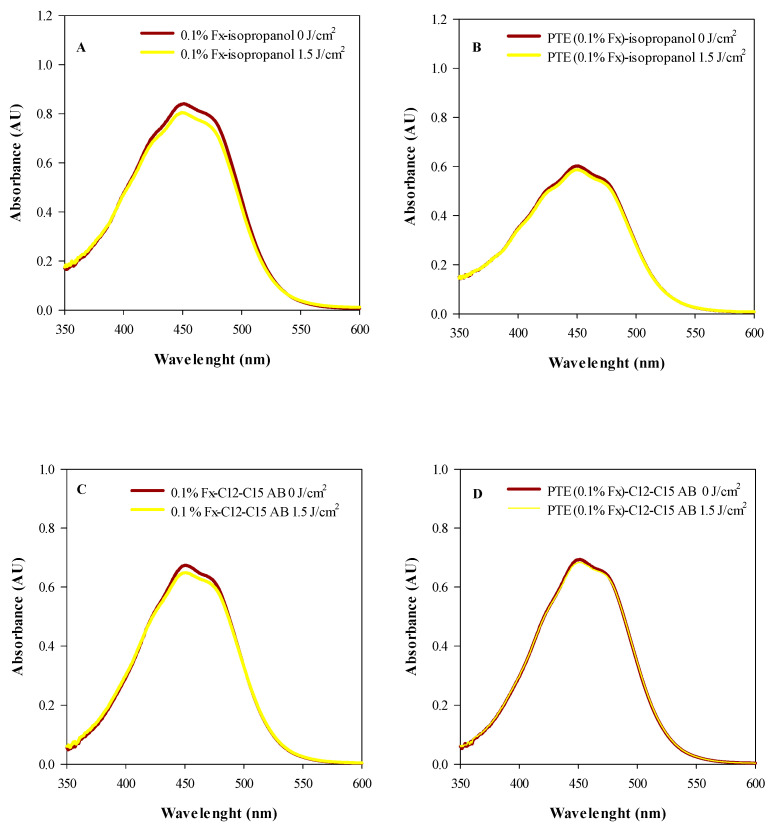
Photostability studies based on the electromagnetic spectra of tested samples irradiated (yellow line) or not (red line) with 1.5 J/cm^2^ UVB rays. (**A**) Isopropanol solution of 0.1% fucoxanthin (Fx); (**B**) Isopropanol solution of *Phaeodactylum tricornutum* ethanolic extract (PTE, 0.1% Fx); (**C**) 0.1% Fx dissolved in C12–C15 alkylbenzoate (C12–C15 AB); (**D**) PTE (0.1% Fx) dissolved in C12–C15 alkylbenzoate (C12–C15 AB). Three independent experiments in triplicate (*n* = 3) were carried out for each tested sample.

Before testing the phototoxicity and skin photoprotection of 0.1% Fx and PTE (0.1% Fx) on the reconstructed human epidermidis (RHE) model, photostability studies were performed to investigate the behaviour of them following exposure to UVA and UVB radiation (Figure 4 and Figure 5, respectively).

Indeed, in order to be effective, treatments must be, first of all, as photostable as possible following exposure to the same doses of radiation then used to evaluate their effectiveness. In this regard, both isopropanolic solutions of 0.1% Fx and PTE (0.1% Fx) and the same treatments dissolved in C12–C15 alkylbenzoate, were evaluated.

As it is possible to observe from Figure 2, the two isopropanol solutions of PTE (0.1% Fx) (Figure 2A) and 0.1% Fx (Figure 2B), already appear quite photostable at the UVA dose recommended for phototoxicity studies (6 J/cm^2^), with a degradation of 8.44% and 17%, respectively. However, interestingly, by conveying PTE (0.1% Fx) and 0.1% Fx in C12–C15 alkylbenzoate, and exposing them to an UVA radiation dose which simulates the solar radiation (26 J/cm^2^), both treatments showed exceptional photostability, with a negligible degradation rate equal to 0.23% and 0.8%, respectively (Figure 4C,D, respectively).

A similar behaviour was also found after exposure to UVB rays (1.5 J/cm^2^). In fact, as can be seen from Figure 5, the isopropanol solutions 0.1% Fx (Figure 5A) and PTE (0.1% Fx) (Figure 5B) showed excellent photostability, showing only a very slight photodegradation equal to 3.18% and 1.60%, respectively. According to what has been observed with UVA radiation, when 0.1% Fx and PTE (0.1% Fx) were conveyed in C12–C15 alkylbenzoate and exposed to the same dose of UVB radiation, the photodegradation rate decreased, reaching negligible values equal to 0.65% (Figure 5C) and 0.19% (Figure 5D), respectively.

#### 2.3.3. Phototoxicity and Skin Photoprotection Studies

Once the photostability of the treatments was verified, the potential phototoxicity of the latter was evaluated on the RHE model. This model, thanks to the presence of the stratum corneum, can predict eventual phototoxic effects on the human epidermis with an excellent transability of the results from the in vitro to in vivo scenario. Consequently, under appropriate test conditions, a negative result in a 3D skin model, such as this, indicates that the acute photo-irritation potential of the formulation can be considered low.

**Figure 6:**
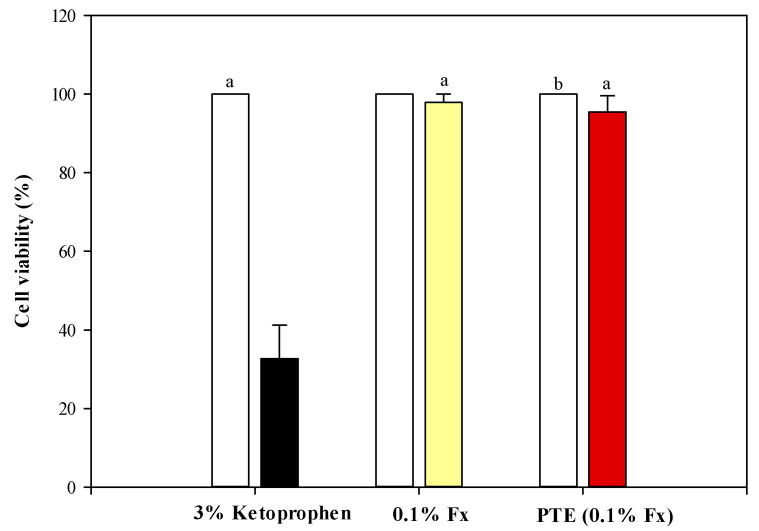
MTT assay on reconstructed human epidermidis (RHE) models treated with 3% ketoprofen (positive control, dark bar), 0.1% fucoxanthin (0.1% Fx, yellow bar), and *Phaeodactylum tricornutum* ethanolic extract (PTE) containing 0.1% Fx (PTE (0.1% Fx), red bar), all dissolved in C12–C15 alkylbenzoate, irradiated or not (white bars, negative control) with 6 J/cm^2^ UVA rays. Three independent experiments in triplicate (*n* = 3) were carried out for each tested sample. ^a^ *p* < 0.05 vs. 3% ketoprofen; ^b^ *p* < 0.05 vs. PTE (0.1% Fx).

Figure 6 shows the results of the cell viability percentage (%) detected after the treatment with 3% ketoprofen (dark bar), known phototoxic compound as positive control, 0.1% Fx (yellow bar), and PTE (0.1% Fx) (red bar), all dissolved in C12–C15 alkylbenzoate, with respect to their negative controls, which consist of the same treatments without 6 J/cm^2^ UVA irradiation (white bars).

Ketoprofen, as expected, showed a marked and statistically significant (*p* < 0.05) phototoxicity, reducing the cell viability by 67.29% compared to the negative control (Figure 6). On the contrary, both 0.1% Fx and PTE (0.1% Fx) showed a slight, but not statistically significant, decrease in the cell viability (2.14% and 4.53%, respectively) with respect to the non-irradiated controls (Figure 4), thus showing an excellent safety profile.

After this, photoprotective studies were carried out on the same RHE model in order to verify the efficacy of Fx and PTE as skin photoprotective agents against the cytotoxic, oxidative and inflammatory events induced by both UVA and UVB radiations.

At this purpose, RHE models were treated with C12–C15 alkylbenzoate only, 0.1% Fx and PTE (0.1% Fx), both dissolved in C12–C15 alkylbenzoate, and irradiated or not with dose of UVA and UVB rays which simulate the solar radiation (26 J/cm^2^ and 1.5 J/cm^2^, respectively).

**Figure 7:**
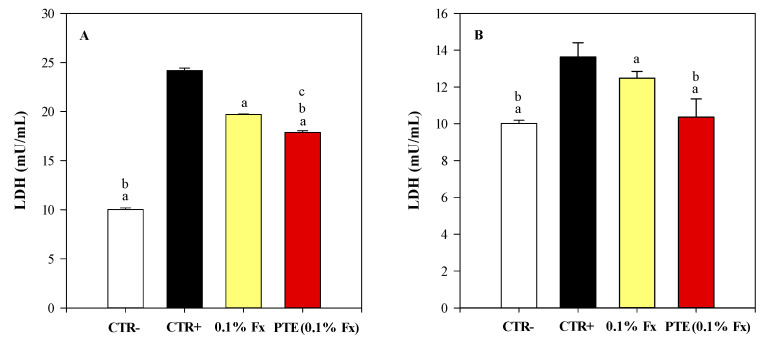
Lactate dehydrogenase (LDH) activity recorded on reconstructed human epidermidis (RHE) tissues culture media irradiated with 26 J/cm^2^ UVA rays (**A**) and 1.5 J/cm^2^ UVB rays (**B**). Negative control (CTR−, white bar) refers to RHE models treated with the sunscreen carrier (C12–C15 alkylbenzoate) but not irradiated. On the contrary, positive control (CTR+, dark bar), 0.1% Fucoxanthin (0.1% Fx, yellow bar), and *Phaeodactylum tricornutum* ethanolic extract (PTE) containing 0.1% Fx (PTE (0.1% Fx), red bar) refer to RHE models treated with the sunscreen carrier (C12–C15 alkylbenzoate), 0.1% Fx and PTE (0.1% Fx), respectively, and irradiated. Three independent experiments in triplicate (*n* = 3) were carried out for each tested sample. ^a^ *p* < 0.05 vs. CTR+; ^b^ *p* < 0.05 vs. 0.1% Fx; ^c^ *p* < 0.05 vs. CTR−.

Figure 7 shows the results related to the lactate dehydrogenase (LDH) activity detected in RHE culture media treated with C12–C15 alkylbenzoate (CTR+, dark bar), 0.1% Fx (yellow bar), and PTE (0.1% Fx) (red bar), and irradiated with UVA (Figure 7A) and UVB (Figure 7B) rays. White bar represents the negative controls (CTR−), that are RHE models treated with C12–C15 alkylbenzoate and not irradiated (Figure 7). Both UVA and UVB doses, significantly affected the cell viability increasing the LDH activity by 2.41-fold and 1.36-fold compared to the CTR− (*p* < 0.05) after exposure to UVA and UVB rays, respectively (Figure 7A,B, respectively). Both 0.1% Fx and PTE (0.1% Fx) were able to significantly (*p* < 0.05) decrease the LDH activity compared to CTR+, showing a reduction of 1.23-fold and 1.35-fold compared to the CTR+ after exposure to UVA radiation (Figure 5A), and by 1.11- and 1.28-fold after exposure to UVB radiation (Figure 7B). Finally, interestingly, a statistically significant difference was always recorded between 0.1% Fx and PTE (0.1% Fx) (*p* < 0.05), with the latter treatment showing the greatest efficacy in reducing LDH activity, even restoring, after UVB irradiation, the LDH values of the CTR− (Figure 7B).

**Figure 8:**
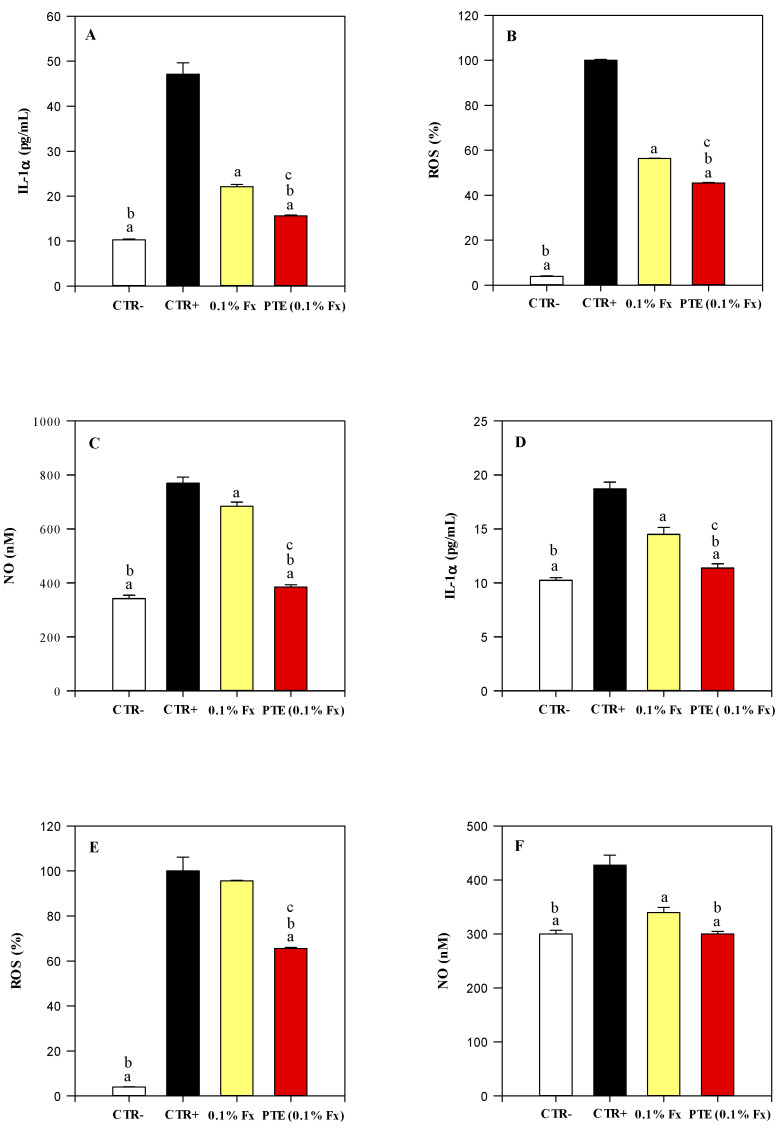
Dosage of antioxidant and anti-inflammatory markers on reconstructed human epidermidis (RHE) tissues and culture media irradiated with 26 J/cm^2^ UVA rays (**A**–**C**) and 1.5 J/cm^2^ UVB rays (**D**–**F**). Negative control (CTR−, white bar) refers to RHE models treated with the sunscreen carrier (C12–C15 alkylbenzoate) but not irradiated. On the contrary, positive control (CTR+, dark bar), 0.1% Fucoxanthin (0.1% Fx, yellow bar) and *Phaeodactylum tricornutum* ethanolic extract (PTE) containing 0.1% Fx (PTE (0.1% Fx), red bar) refer to RHE models treated with the sunscreen carrier (C12–C15 alkylbenzoate), 0.1% Fx and PTE (0.1% Fx), respectively, and irradiated. Three independent experiments in triplicate (*n* = 3) were carried out for each tested sample. ^a^ *p* < 0.05 vs. CTR+; ^b^ *p* < 0.05 vs. 0.1% Fx; ^c^ *p* < 0.05 vs. CTR−.

The UVA and UVB photoprotective efficacy of 0.1% Fx and PTE (0.1% Fx) was also evaluated by monitoring the release of nitric oxide (NO) and interleukin (IL)-1α, as well as the scavenging capacity against oxygen free radicals (ROS) (Figure 8).

Figure 8A–F show the results of the three markers investigated recorded after UVA and UVB irradiation, respectively.

UVA and UVB radiations increased significantly (*p* < 0.05) the IL-1α (4.6 times and 1.83 times, respectively) and NO (2.25 times and 1.42 times, respectively) release in the CTR+ with respect to the CTR− (Figure 8A,C, and Figure 8D,F, respectively). In both cases, 0.1% Fx and PTE (0.1% Fx) significantly reduced (*p* < 0.05) the IL-1α (2.08-fold and 2.88-fold, respectively, after UVA irradiation, and 1.36-fold and 1.73-fold after UVB irradiation) and NO release (1.13-fold and 2-fold, respectively after UVA irradiation, and 1.26-fold and 1.42-fold after UVB irradiation) with respect to CTR+.

Moreover, 0.1% Fx and PTE (0.1% Fx) were able to scavenge the ROS by 43.68% and 54.56% after exposure to UVA rays, and by 4.34% and 30.13% after exposure to UVB rays with respect to the CTR+.

Interestingly, also in this case, according to the previous results, PTE (0.1% Fx) shows a powerful and statistically significant (*p* < 0.05) antioxidant and anti-inflammatory activity than 0.1% Fx (Figure 8A–F), confirming, once again, the greater photoprotective activity of the plant-complex with respect to the pure molecule tested at the same concentration present within the PTE.

The RHE tissues treated with 0.1% Fx and PTE (0.1% Fx) and irradiated or not with 26 J/cm^2^ UVA and 1.5 J/cm^2^ UVB doses, were analysed also from histological point of view.

**Figure 9:**
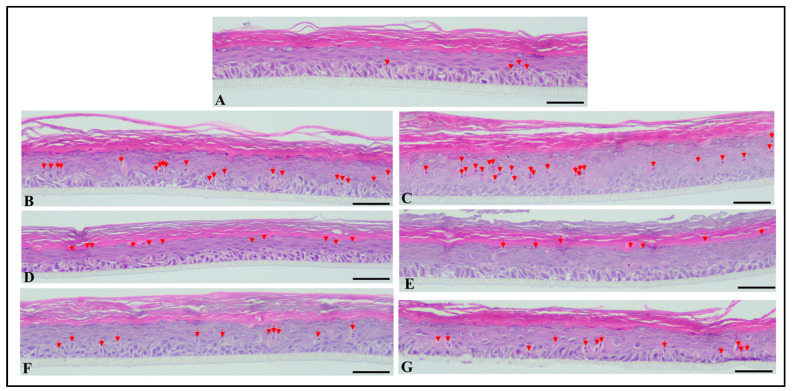
Microphotographs of RHE tissues, stained with haematoxylin and eosin—red arrows show apoptotic/sunburn cells. (**A**) Normal non-damaged RHE control samples showing few apoptotic/sunburn cells. (**B**,**C**) RHE control tissues exposed to UVA (**B**) and UVB (**C**) with significantly increased number of apoptotic and sunburn cells. (**D**,**E**) RHE tissues exposed to UVA (**D**) and UVB (**E**) and previously treated with *Phaeodactylum tricornutum* ethanolic extract (PTE) containing 0.1% Fx showing significantly reduced number of apoptotic/sunburn cells compared to UVA and UVB exposed, un-treated samples. (**F**,**G**) RHE tissues exposed to UVA (**F**) and UVB (**G**) and previously treated with 0.1% Fucoxanthin (Fx) showing significantly reduced number of apoptotic/sunburn cells compared to UVA and UVB exposed, un-treated samples. Scale bar = 50 µm.

The mean number of apoptotic cells was 4.4 in a linear extension of tissue measuring 500 µm in the control samples, while in control samples exposed to UVA and UVB radiations, the mean number of apoptotic/sunburn cells increased significantly (*p* < 0.05) to 20 and 20.6 per 500 µm, respectively. Samples exposed to UVA and UVB rays, and previously treated with PTE (0.1% Fx) showed a mean of 10.2 and 8.8 apoptotic/sunburn cells per 500 µm, respectively, whereas samples treated with 0.1% Fx showed a mean of 10.2 and 11 apoptotic/sunburn cells per 500 µm, respectively. These values were significantly higher compared to those obtained with the non-irradiated negative controls (*p* < 0.05), but, at the same time, significantly lower with respect to the UVA and UVB irradiated positive controls (*p* < 0.05) (Figure 9).

No statistically significant difference was observed between PTE (0.1% Fx) and 0.1% Fx, independently from the UVA or UVB exposition, nor between control samples exposed to UVA or UVB rays.

**Figure 10:**
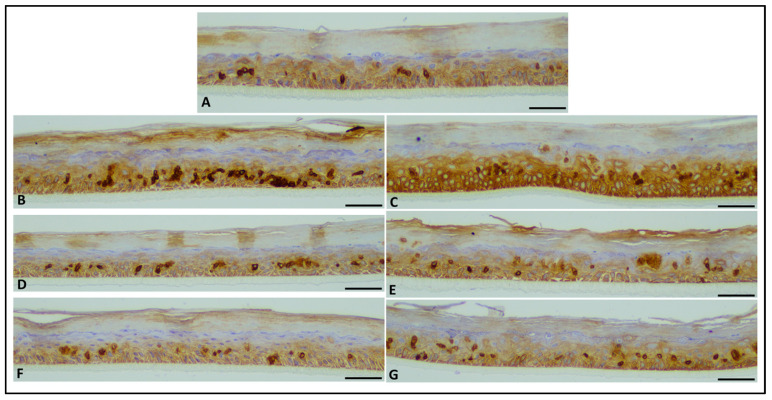
Microphotographs of RHE tissues, immunostained with anti-CK5&6. (**A**) Normal non-damaged RHE control samples showing mild expression in scattered cells. (**B**,**C**) RHE control tissues exposed to UVA (**B**) and UVB (**C**) showing high expression compared to control non-exposed samples. (**D**,**E**) RHE tissues exposed to UVA (**D**) and UVB (**E**) and previously treated with *Phaeodactylum tricornutum* ethanolic extract (PTE) containing 0.1% Fx showing moderate expression compared to UVA and UVB exposed, untreated samples. (**F**,**G**) RHE tissues exposed to UVA (**F**) and UVB (**G**) and previously treated with 0.1% Fucoxanthin (Fx) showing moderate expression compared to UVA and UVB exposed, un-treated samples. Scale bar = 50 µm.

Cytokeratin 5&6 identified intensely immunostained apoptotic/sunburn cells on a background of diffuse mild expression in the basal layers, and showed mild expression in non-irradiated controls, while expression was high in the UVA and UVB rays exposed controls, and moderate in both PTE (0.1% Fx) and 0.1% Fx-treated RHE tissues exposed to UVA or UVB radiations (Figure 10).

**Figure 11:**
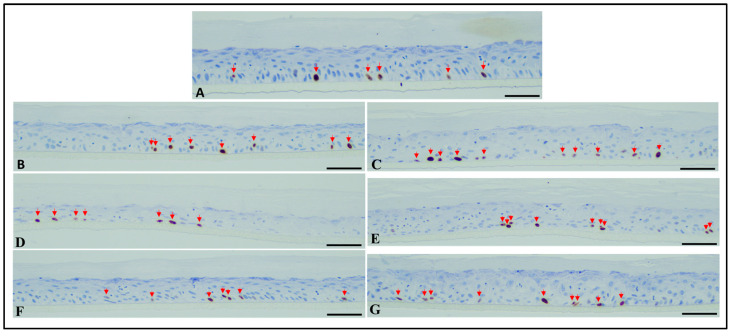
Microphotographs of RHE tissues, immunostained with anti-Ki67. No significant differences in proliferative cells between all studied samples were observed (red arrows show Ki67-positive proliferating cells). (**A**) Normal non-damaged un-treated RHE controls. (**B**,**C**) RHE tissues exposed to UVA (**B**) and UVB (**C**). (**D**,**E**) RHE tissues exposed to UVA (**D**) and UVB (**E**) and previously treated with *Phaeodactylum tricornutum* ethanolic extract (PTE) containing 0.1% Fx. (**F**,**G**) RHE tissues exposed to UVA (**F**) and UVB (**G**) and previously treated with 0.1% Fucoxanthin (Fx). Scale bar = 50 µm.

On the contrary, no difference in the number of the proliferative cells, detected by using the Ki67 as proliferation marker, was observed between the non-irradiated control samples, controls exposed to UVA and UVB rays and RHE tissues exposed to UVA or UVB radiations treated with PTE (0.1% Fx) and 0.1% Fx (all values ranging from 21 to 27 per 1500 µm, and mean values ranging from 7 to 9 per 500 µm) (Figure 11).

## 3. Discussion

Microalgae, comprising over 72,500 species and 16 classes belonging to the three major groups of Chlorophyceae, Chrysophyceae, and Diatoms, are unicellular photosynthetic organisms, living in saline or freshwater ecosystems [17]. Several countries are involved in the microalgae research and cultivation to obtain high-value products, with an annual production of biomass of 19,000 tons and a turnover of about 5.7 billion dollars [18,19]. Diatoms represent the most heterogeneous microalgae group, counting about 20,000 different species and very different dimensions (from few microns to few millimetres) and symmetry, which can be bilateral or radial leading to pennate or centric specimens [20]. Moreover, they are generally surrounded by a silica skeleton, namely frustule, and show a peculiar pigment profile expressing, unlike land plants and green algae, chlorophyll a and c instead of chlorophyll a and b, other than xanthophyll-type carotenoids, which give it the typical golden-brown colour [20]. Trimeric and oligomeric complexes of xanthophyll-chlorophyll-proteins located on the thylakoids of diatoms are involved in the blue-green region light harvesting [21]. Thanks to these particular features, these microalgae are widely used as a model to investigate photosynthesis and photoprotective mechanisms [20]. Microalgae are great sources of nutrient and non-nutrient compounds such as proteins, polysaccharides, lipids, fatty acids, pigments, vitamins, and minerals, with several and well-known health effects [17]. Several cultivation methods have been developed to enhance these products accumulation [22,23,24,25], but the pigments remain, thanks to their ecological relevance, as well as their wide spectrum of health applications, the most investigated [26].

Microalgae contain several visible light-harvesting complexes, such as chlorophylls, carotenes, xanthophylls, and phycobiliproteins. In this manuscript, which had the aim of studying the diatom *P. tricornutum,* we will discuss the carotenoids, the pigments most expressed by this group of microalgae. Carotenoids are yellow-orange-red C-40-polyene complexes that can be divided, according to the presence or absence of one or more oxygens in their structure, into carotenes and oxy-carotenes, also known as xanthophylls. The most recognized and largely produced algae xanthophylls are lutein, astaxanthin, canthaxanthin, and fucoxanthin [17]. Although these pigments are produced during the vegetative phase, as they are responsible for light-harvesting, it is known that their production can be influenced by various biotic and abiotic factors such as light intensity and duration, nutrient availability, pH, temperature, salinity, and heavy metals [27,28,29,30].

Fx, which represents about the 10% of the total occurring carotenoids, is one of the most effective microalgae carotenoids [31]. The most abundant naturally occurring isomer, and the most biologically active, is the trans one (E-Fx). However, it has been demonstrated that the isomerization may be modified during extraction using, for example, high temperatures [32,33]. It is therefore important to develop adequate extraction methods to obtain a good extraction yield without modify the native phytochemical profile of the microalgae. Despite several papers are available about the health properties of Fx, the microalgal production as well as the pre-treatment and emerging extraction technologies to obtain the best yields in terms of Fx, are still very scarce. Recently, the available literature has been reviewed to shed light on these critical aspects [34].

Pigments can be isolated from microalgal biomass using several methods such as solvent, soxhlet-assisted, enzyme-assisted, and ultrasound-assisted extraction [35]. The extraction method chosen as well as the type of solvents and drying process can greatly influence the yield in bioactive compounds [36]. Di Lena et al. [37] have recently investigated the carotenoids profile of five microalgae industrially produced in outdoor photobioreactors. The investigated microalgae showed a species-specific carotenoids profile, although a xanthophylls prevalence with respect to carotenes, were always detected. β-Carotene was the only common carotenoid to all species, with the lowest concentration detected in *P. tricornutum*. On the contrary, this species showed the second highest total carotenoid content (1.02 g/100 g dry extract). (E)-Fx is the most abundant compound (0.78 g/100 g dry extract), accounting for about 76% of the total detected carotenoids. Despite the results of the present study, from the qualitative point of view, are quite similar (E-Fx 77.45%), the quantification of (E)-Fx in the PTE results about nine-fold higher (6.67 g/100 g PTE) [37]. According to our results, (9Z)-Fx and (13Z)-Fx, as well as diadinoxanthin and diatoxanthin were also detected in *P. tricornutum*. These two last metabolites play a pivotal role in specific molecular responses to stressful light conditions, allowing the thermal dissipation of excess light energy and providing effective photoprotection to the photosynthetic apparatus of algae and diatoms. As in the present study, authors did not detect violaxanthin, antheraxanthin and zeaxanthin, xanthophylls that normally accumulate in diatoms during long-term exposure to bright light [38], indirectly demonstrating that the light exposure used in the photobioreactor was not stressful for *P. tricornutum,* both for the present study and the previous one. Another study has evaluated two new extraction methods, pressurized liquid extraction (PLE) and microwave-assisted solvent extraction (MAE) for the recovery of bioactive compounds from *P. tricornutum* [39]. Extraction yield (% *w*/*w*), total phenolic content, carotenoids, and chlorophylls, as well as the antioxidant activity recorded by TEAC assay, were the parameters considered to make a comparison between the different extraction methods. Time and temperature have been also considered as critical parameters. Moreover, authors made a detailed chemical characterization of pigments by HPLC–DAD–MS/MS analysis. In both cases, the main detected compounds belonged to the class of carotenoids, with (E)-Fx as the most abundant compound. However, the highest extraction yield was obtained with PLE, the extraction method most similar to that used in the present study. Again, two trans isomers of fucoxanthin were found, but tentatively identified this time as (13Z)-Fx and (13′Z)-Fx. With respect to our study and the previous one carried out by Di Lena et al. [37], the authors identified, among other carotenoids, only diatoxanthin, whereas three other peaks, which the authors tentatively attribute to carotenoids class, remain unidentified. According to our study, Gilbert-López et al. [39] identified also several pigments belonging to the chlorophylls class, in particular chlorophyll a and c-types. The Fx content was quantified also in this case reaching, following PLE at the optimal extraction, 0.77 g/100 g algae. Even if the results are not always expressed in the same way, as some authors tend to express them in terms of algal biomass, others instead as dry extract, there is no doubt that the cultivation and extraction conditions massively affect the pigment yield and, in particular, the Fx content of the microalgae, and that some strategies, such as the purification step used in the present study, can greatly enrich the extract in xanthophylls. Furthermore, the use of a multidisciplinary approach, such as that used in the present study, allows to monitor, also in progress, the growth behaviour and metabolism of the microalga, with particular emphasis, in the specific case of *P. tricornutum*, to mixotrophy capable of favouring an overproduction of carotenoids.

These pigments possess several functional health properties such as antioxidant, anti-inflammatory, and skin photoprotective [7,40,41,42,43,44,45,46]. In vitro and in vivo studies highlighted both direct and indirect antioxidant and anti-inflammatory activity, through the modulation of specific genes and proteins [42]. Unlike other carotenoids, Fx, thanks to their particular structure, are a nucleophile of choice, able to scavenge various types of free radicals such as trichloromethylperoxyl, oxygen, and nitrogen radicals [43]. The antioxidant activity of Fx has been evaluated in vitro by several cell-free assays such as 2,2-diphenyl-1-picrylhydrazyl (DPPH) assay, FRAP, and TEAC [47,48,49,50,51]. A concentration-dependent behaviour was always observed, according to our results, with similar IC_50_ values, which range, depending on the specific test, between 30 and 200 µg/mL [47,48,49,50,51,52]. In addition, it has been demonstrated that Fx, unlike other carotenoids, quenches free radicals also in anoxic conditions [53]. The most naturally occurring trans isomer showed stronger activity than the cis isomer, although pure Fx has been shown to be susceptible to oxidation, whereas this event does not occur when the bioactive compound is within a plant-complex [54]. This could be the reason why, although Fx represents the most abundant compound in PTE, in our study the plant-complex always shows a biological activity, be it antioxidant, anti-inflammatory, and skin photoprotective, greater than the pure molecule tested at the same concentrations within PTE. The antioxidant properties of Fx were investigated also on several cell models such as HaCaT, ARPE-19, and RAW264.7, by which it has been demonstrated that it can significantly decrease the oxidative stress and repair DNA damage, reverting also the cell morphological changes [45,48,49,52]. However, as specified above, in addition to the direct antioxidant activity such as free radical scavenging activity, Fx is also capable to exert its antioxidant activity by modulating several signalling pathways such as the nuclear factor erythroid 2–related factor/antioxidant response element (2Nrf2/ARE), extracellular signal-regulated kinase (ERK)/p38, phosphatidylinositol 3-kinases/protein kinase B (PI3 K/Akt), and sirtuin 1 (Sirt1). Moreover, it may modulate production or expression of ROS, glutathione (GSH), glutathione S-transferase (GST), catalases, HO-1, NQO1, and apoptosis-related proteins [48,51,55,56,57,58,59]. Together with oxidative stress, inflammation, with an over-production of pro-inflammatory mediators, plays a key role in the onset of various disorders [50,60,61]. Bioactive pigments from microalgae act on several signalling pathways related to inflammation. From this point of view, Fx is the most investigated microalgal pigment. There are several in vitro and in vivo studies which demonstrate its effectiveness [45,49,60,61,62,63]. Specifically, it has been demonstrated that Fx may inhibit the production of prostaglandins (PGE_2_) and NO in a concentration-dependent manner, by down-regulating the expression of COX-2 and iNOS, respectively, as well and the activation of NF-κB via IκB-α mediated phosphorylation inhibition. Moreover, Fx significantly reduced the phosphorylation of ERK1/2, p38 and Jun N-terminal kinases (JNK) mitogen-activated protein kinases (MAPKs), causing a down-regulation of MAPKs in lipopolysaccharide (LPS)-stimulated RAW 264.7 cells [62], TNF-α stimulated HaCaT cells and LPS-stimulated THP-1 macrophages and Hepa1-6 cells, where Fx significantly decrease the release of several inflammatory markers such as TNF-α, MCP-1, IL-1, and IL-6 [49,50].

The antioxidant and anti-inflammatory mechanisms highlighted above, together with the promotion of skin barrier formation through the induction of UV-sensitive gene expression such as filaggrin, play a pivotal role also in the skin photoprotective activity of Fx, that it has been demonstrated by various in vitro methods and in vivo models [64,65,66]. On the other hand, skin photoprotection studies carried out on plant-complexes obtained from microalgae are very scarce and this is the first study performed on *P. tricornutum*. Rodriguez-Luna and coworkers [49] evaluated, both in vitro and in vivo, the anti-inflammatory and antioxidant properties of a Fx-based topical formulation, showing that it can act as effective photoprotective agent against UV rays, modulating the inflammatory response via the COX-2 and iNOS down-regulation, and HO-1 protein up-regulation via the Nrf-2 pathway. Recently, Tavares and coworkers [16] assessed the photostability and phototoxicity of Fx isolated from the alga *Desmarestia anceps* in 3T3 mouse fibroblast cells and full-thickness reconstructed human skin (RHS) as well as its ability to inhibit UVA-induced ROS formation on keratinocytes and RHS. Furthermore, they tested the antioxidant properties of a sunscreen formulation containing 0.5% Fx onto the same RHS model. Contrary to what has been observed in the present study, at the same UVA dose (6 J/cm^2^), the authors observed a certain photo-instability of Fx (35% UVA absorbance reduction), whereas a low photodegradation was recorded when Fx was incorporated in the sunscreen formulation (12.5% UVA absorbance reduction). This could be due to the higher concentration of Fx used, 5 times higher than that used in the present study, which could exert pro-oxidant effects due to the high reactivity of the molecule. On the contrary, according to our results, authors did not observe any phototoxicity on the RHS model, on which Fx it has proved to be also particularly effective in contrasting ROS formation. However, one of the limitations of the above study and of many others present in the literature compared to this is represented by the use of only UVA or UVB radiation to evaluate the phototoxic and skin photoprotective effect of a molecule or a plant-complex.

Indeed, although solar UV radiation consists mainly of UVA rays, it is supposed that these radiations play a pivotal role only in the photoaging process, being a potent driver of oxidative free radical damage to DNA and other macromolecules [67]. On the contrary, UVB rays can penetrate much deeper in the skin, are potent inflammatory stimuli causing the release of numerous cytokines, and are able to interact directly with DNA, inducing mutagenic photolesions that can lead to mutations such as thymine dimers [67]. These mutations, if the UVB exposition is not too protracted over time, can be conveniently repaired by DNA repair systems. Conversely, apoptotic pathways mediated by p53 protein, which upregulate several apoptosis-promoting genes such as Bcl-2 Associated X-protein (Bax), Fas/Apo-1, or down-regulate apoptosis-suppressing genes such as Bcl-2, are activated to eliminate damaged cells [68]. In addition, it has been demonstrated that p53 protein can activate the Fas gene, which plays a pivotal role in the induction of the major acute clinical effects of UV irradiation on normal human skin, which are the sunburn inflammation and tanning [68]. Leaving out the second aspect, which is outside the topics covered in this article, the histological changes following UV irradiation include thickening of the stratum corneum, epidermis, and dermis, as well as intercellular and perivascular swelling. According to our results, sunburn erythema is the most conspicuous and well recognized acute cutaneous response to the UV irradiation [68]. The action spectrum of erythema is consistent with the hypothesis that inflammation and UV interactions with DNA play a pivotal role, whereas the indirect oxidative damage might also occur secondarily to endogenous photosensitization [68]. According to what has been observed in the present study, UV radiations and in particular UVB rays lead to the formation of the most of sunburn cells, easily recognizable by the pyknotic nuclei and their eosinophilic cytoplasm, as revealed by haematoxylin/eosin staining [69]. It has been demonstrated that they can be observed in suprabasal regions already 30 min after irradiation, reaching the maximum density 24 h later, at which time they are also visible in the middle and upper dermis [69]. Furthermore, according to our results, it has been demonstrated that in human skin, UV radiation affects cytokeratins and adhesion molecules as well as other components of the cytoskeleton involved in the control of normal cell growth [70]. Considering this, in order to properly evaluate the photostability and skin photoprotective properties of a bioactive compound or a plant-complex, it is important to evaluate its behaviour after exposure to both UVA and UVB radiation at doses simulating the solar radiation. This allows to simulate in vitro as much as possible what occurs in vivo and ensure greater transferability of the observed results.

In summary, the complex of our data consistently shows that the PTE, highly rich in Fx, has remarkable skin photoprotective properties. The potential relevance of this result resides in the interest that microalgae pigments are attracting for the development of skin photoprotective products, as shown by different preparations present in the market (see Table S1 in Supplementary). Some of these products contain xanthophylls such as astaxanthin and zeaxanthin, but not Fx. Conversely, our study demonstrates that also a Fx-rich plant-complex can realize an excellent photoprotection. Hence, our data suggest that *P. tricornutum* can be considered a possible source for new, fucoxanthin-based skin photoprotective products.

## 4. Materials and Methods

### 4.1. Culture and Growth Conditions of P. tricornutum

Specimens of PT, originally isolated from a seaside resort in Blackpool, England, were obtained from Culture Collection of Algae and Protozoa (CCAP, SAMS Ltd., Dunbeg, Scotland, UK), with the number CCAP1055/1. The cultivation medium, Artificial Sea Water (ASW) consisting of 30 g/L of Tropic Marin^®^ Classic (Dr. Biener GmbH, Wartenberg, Germany) enriched with the f/2 + Si Guillard’s medium for diatoms, is composed by the following salts per litre: NaNO_3_ 800 mg, NaH_2_PO_4_ 60 mg, Na_2_EDTA 0.8 mg, FeCl_3_ 0.6 mg, CuSO_4_ 125 μg, ZnSO_4_ 440 μg, CoCl_2_ 125 μg, MnCl_2_ 3 mg, Na_2_MoO_4_ 108 mg, cyanocobalamin (Vitamin B12) 1 μg, biotin 1 μg, thiamine HCl (Vitamin B1) 200 μg, and sodium metasilicate (Na_2_SiO_3_) 300 mg (all reagents were from Sigma-Aldrich and were used without further purification) [71]. The final pH was adjusted to 8.0 with 1M NaOH or 1M HCl prior to autoclaving at 15 psi for 15 min.

At the laboratory scale, PT was cultured for 14 days in Erlenmeyer flasks with breathable cotton cap, containing 10% medium, on a rotary flask shaker at 150 rpm, 25 °C, and light intensity of 250 μmol m^−2^ s^−1^. The nitrate content of the culture was monitored through a colorimetric strip (Merck KGaA, Darmstadt, Germany) to check its consumption in the medium. The cell concentration was monitored through cell count with a Thoma chamber.

To obtain a substantial quantity of algal biomass to process and extract, PT was cultivated in large scale at the Archimede Ricerche s.r.l. production plant (Camporosso, IM, Italy), inside naturally illuminated bioreactors. The cultivation conditions in this setting were a replica of the laboratory scale, except for the introduction of air lift bubbling (0.5 vvm) to keep the culture suspended with air enriched with 1% CO_2_. Harvested algal biomass, which reached a concentration of 1 g/L, was freeze-dried (Criofarma s.a.s, TO, Italy), milled, and kept at −20 °C before the extraction.

### 4.2. Extraction Procedure

A two-step solvents extraction method was used to recover the pigments from the microalgal freeze-dried biomass. First, the biomass was extracted with 100% ethanol for 24 h at room temperature (RT). After this, to remove the excess of chlorophylls, enological coal was added (1:100, *w*/*w*). The mixture was then mixed for 1 h at RT and subsequently filtered through filter paper to remove the coal and the exhaust biomass. The resulting extract was evaporated to dryness, and then recovered in a mixture of water and ethyl acetate (1:1, *v*/*v*) to remove salts and to suspend the apolar fraction. The suspension was shaken for 30 min at 150 rpm and then allowed to settle for 30 min. The supernatant was collected and the process was repeated two more times. After the final wash, the ethyl acetate fraction, rich in carothenoids, was evaporated under vacuum at 40 °C to obtain the apolar fraction. The dried apolar fraction was then suspended in food-grade ethanol to obtain the final formulation consisting of 70:30 (*w*/*v*) extract/ethanol. The latter was stored at 4 °C until analyses. The total yield of final extract (*w*/*w*) ranges between 5–15% depending on the biomass humidity, ashes and carotenoid content.

### 4.3. Transmitted Light and Fluorescence Microscopy

Light microscope observations were performed under a transmission light and epifluorescence Leica DM 2000 microscope, coupled with a DFC 320 camera (Leica Microsystems, Wetzlar, Germany).

For the observation of neutral lipids within diathom cells the BODIPY^TM^ 505/515 (4,4-difluoro-1,3,5,7-tetramethyl-4-bora-3a,4a-diaza-s-indacene) fluorescent dye was used (cat. D3921, Thermo Fisher Scientific, Waltham, MS, USA). Aliquots of 100 µL diatom cell suspensions (1 × 10^6^ cells/mL) were incubated for 7 min in the dark with a final concentration of 0.1 µg/mL BODIPY^TM^ in culture medium, obtained from a stock solution of 0.5 mg/mL BODIPY^TM^ in DMSO. After staining, cells were spun down, washed with culture medium, and wet mounted on a microscope slide. Microscope observations were performed with the above microscope, at the following excitation and emission wavelengths: 488 nm and 510 nm, respectively.

### 4.4. SEM Microscopy

For SEM observations, samples of fresh PT culture were treated following the method reported by Veltkamp et al. [72]. The fresh culture was rapidly washed in distilled water to remove salt excess, centrifuged, washed with a 3-fold volume of absolute ethanol at −18 °C to allow for mild dehydration, incubated at −18 °C for a time length proportional to the sample volume, and then stored at 4 °C overnight. Freezing at −18 °C and overnight refrigerator process were repeated the following day, as well before drying the algal biomass. Samples were then placed onto Whatman 22 μm filters, dehydrated in an ascending series of ethanol washes (50–80–90–100%), and air-dried according to Balbi et al. [73] with some modifications. Finally, small portions of filters were mounted on stubs using two-sided adhesive carbon tape and sputtered with a 10-nm layer of gold. The samples were examined using a SEM VEGA3-Tescan-type LMU microscope (Apollo, Tescan USA Inc., Cranberry Twp, PA, USA), operating at an accelerating voltage of 20 kV.

### 4.5. Phytochemical Characterizaztion

The phytochemical profile of PTE was determined by HPLC-DAD-APCI-MS/MS (1100 series, Agilent technologies Inc., Santa Clara, CA, USA) according to Lopez et al. [39], with some modification. Chomatografic separation was carried out at 25 °C using a Luna C18(2) 100 Å 250 mm × 4.6 mm, 5 μm (Phenomenex, Torrance, CA, USA) column with a mobile phase consisting of methanol–MTBE–water (90:7:3, *v*/*v*/*v*) (solvent A) and methanol–MTBE (10:90, *v*/*v*) (solvent B), according to the following elution program: 0 min, 0% B; 20 min, 30% B; 35 min, 50% B; 45 min, 80% B; 50 min, 100% B; 60 min, 100% B; 62 min, 0% B; 70 min, 0% B. The flow rate was 0.8 mL/min, while the injection volume was 10 μL. The detection was performed at 280, 450 and 660 nm, recording the UV-Vis spectra from 190 to 770 nm. The setting parameters of the mass spectrophotometer, an ion trap 6320 (Agilent Technologies, Santa Clara, CA, USA), were set as follows: positive ionization mode, −3.5 kV capillary voltage, 60 psi nebulizer gas pressure, 350 °C drying temperature, 400 °C vaporizer temperature, 5 L/min drying gas flow and 4000 nA corona current. Acquisition was carried out in full-scan mode (90–1500 *m*/*z*). Automatic MS/MS analyses were also performed setting the fragmentor amplitude to 1 V. Data were acquired by Agilent ChemStation software version B.01.03 and Agilent trap control software version 6.2. Identification was carried out by comparing the retention times, UV–Vis and MS spectra of each analyte with those of commercially available standards (see Table 1), the literature data and UV–Vis and mass spectra databases. The LC-DAD chromatogram acquired at 450 nm was used for relative quantification, expressing the results as mean area percentage (%) ± standard deviation (S.D.) of three independent analyses in triplicate (*n* = 3) with respect to the total area of identified and unidentified compounds. Fx quantification was carried out using an external calibration curve (1–100 µg/mL, R^2^ = 0.9999) built with HPLC-grade analytical standard (purity ≥ 95%) (Merck KGaA, Darmstadt, Germany). Results were expressed as g of Fx/100 g of PTE.

### 4.6. Antioxidant Activity

The antioxidant activity of PTE and Fx, tested at the same concentrations within PTE, was evaluated by several in vitro colorimetric and fluorimetric assays based on different mechanisms and reaction environments. The results, which represent the average of three independent experiments in triplicate (*n* = 3), were expressed as the inhibition percentage (%) of the oxidative/radical activity, calculating the IC_50_ with the respective C.L. at 95% by Litchfield and Wilcoxon’s test using PHARM/PCS software version 4 (MCS Consulting, Wynnewood, PA, USA). All concentration ranges reported refer to the final concentrations of PTE, Fx or reference standard in the reaction mixture.

#### 4.6.1. ORAC Assay

The ORAC assay was carried out according to Cornara et al. [74]. Briefly, 20 μL of PTE (1.5–12.0 μg/mL) and Fx (0.125–1.0 µg/mL) were added to 120 μL of fresh 117 nM fluorescein and incubated for 15 min at 37 °C. Then, 60 μL of 40 mM azobis (2-methylpropionamidine) dihydrochloride (AAPH) were added to trigger the reaction. The fluorescence decay was recorded every 30 s for 90 min by a microplate reader (FLUOstar Omega, BMG LABTECH, Ortenberg, Germany) at the following excitation and emission wavelength: λ_ex_ 485 and λ_em_ 520, respectively. Trolox was used as a reference compound (0.25–2.5 μg/mL), whereas ethanol was used as blank.

#### 4.6.2. TEAC Assay

TEAC assay was carried out according to Bazzicalupo et al. [75], with some modifications. The reagent was prepared by incubating 1.7 mM 2,2′-azino-bis (3-ethylbenzthiazoline-6-sulfonic acid) diammonium salt (ABTS) and 4.3 mM potassium persulfate (5:1, *v*/*v*) for 12 h in the dark at RT. Before to start the assay, the radical solution was diluted to obtain an average absorbance of 0.7 at 734 nm and used within 4 h. Ten microliters of PTE (15–120 μg/mL) and Fx (1.0–8.0) were added to the reagent (200 µL) and incubated at RT for 6 min. The absorbance was recorded at 734 nm, against the same blank reported in the Section 4.6.1, by using an UV–Vis microplate reader (Multiskan GO; Thermo Scientific, Waltham, MA, USA). Trolox was used as a reference compound (1.25–10.0 μg/mL).

#### 4.6.3. FRAP Assay

FRAP assay was carried out according to Occhiuto et al. [76], with some modifications. Briefly, 10 µL of PTE (250–2000 μg/mL) and Fx (20–160 μg/mL) were added to 200 µL of fresh, pre-warmed (37 °C) working reagent consisting of 300 mM buffer acetate (pH 3.6), 10 mM 2,4,6-tris(2-pyridyl)-s-triazine (TPTZ)-40 mM HCl, and 20 mM iron (III) chloride and incubated for 4 min at RT in the dark. The absorbance was recorded at 593 nm using the same instrument and blank reported in Section 4.6.1. Trolox was used as reference compound (1.25–10.0 μg/mL).

#### 4.6.4. BCB Assay

The BCB assay was carried out using a β-carotene emulsion prepared according to Muscarà et al. [77]. Briefly, 0.320 mL of PTE (12.5–100 μg/mL) and Fx (1.25–10 μg/mL) were added to 8 mL of β-carotene emulsion. A β-carotene-free emulsion was used as negative control, whereas a β-carotene emulsion with ethanol (blank) instead of sample was used as positive control. The absorbance was recorded at starting time and during incubation at 50 °C every 20 min for 120 min at 470 nm. BHT was used as reference compound (0.0625–0.5 μg/mL).

### 4.7. In Vitro Cell-Free Photostability Studies

Photostability studies were carried out on PTE (0.1% Fx) and 0.1% Fx isopropanol solutions, as well as on PTE (0.1% Fx) and 0.1% Fx dissolved in C12–C15 alkylbenzoate, one of the most used cosmetic vehicles to solubilize lipophilic bioactive compounds in sunscreens [78]. According to Tavares et al. [16], 1 mL of each sample was added to a 10 mL glass beaker, and then subjected to solvent evaporation by a gentle stream of nitrogen, until a dried film was obtained. PTE (0.1% Fx) and 0.1% Fx organic solutions were subjected or not (negative control) to an UVA and UVB dose of 6 J/cm^2^ and 1.5 J/cm^2^, respectively, to determine their photostability, according to the dose recommended for phototoxicity studies [16]. On the contrary, the formulated samples (PTE 0.1% Fx and 0.1% Fx dissolved in C12-C15 alkylbenzoate) were exposed or not (negative control) to UVA (26 J/cm^2^) [79,80], and UVB (1.5 J/cm^2^) radiations, in order to verify their photostability to a simulated solar radiation [81]. At this purpose, an UV hand-held lamp (RTH469.1, Carl Roth GmbH + Co. KG., Karlsruhe, Germany) equipped with two 15 W UVA (P373.1, Carl Roth GmbH + Co. KG., Karlsruhe, Germany) and UVB (P851.1, Carl Roth GmbH + Co. KG., Karlsruhe, Germany) light tubes, was used. UVA and UVB radiations were exactly dosed using a UV light meter capable of measuring ultraviolet radiation accurately in the range 290–390 nm (PCE-UV34, PCE Italia s.r.l., Lucca, Italy). After irradiation, each sample was suspended, or properly diluted in the case of formulated samples, in 1 mL of isopropanol, and the absorption spectra (280–700 nm) recorded by an UV–Vis spectrophotometer (UV 1601, Shimadzu, Kyoto, Japan). The area under the curve (AUC) of the UV-Vis spectra were recorded using the integration tool of the UVPC Personal Spectroscopy Software Version 3.9 (Shimadzu, Kyoto, Japan) to calculate the photostability of each sample obtained by comparing the mean percentage AUC of three independent experiments in triplicate (*n* = 3) of irradiated samples, with respect to the mean percentage area of three independent experiments in triplicate (*n* = 3) of non-irradiated samples.

### 4.8. Phototoxicity and Skin Photoprotection Studies

#### 4.8.1. In Vitro Reconstructed Human Epidermis Model

In vitro RHE models of 0.50 cm^2^, aged 17 days, were purchased by Episkin (Lyon Cedex, France). This standardized model, histologically similar to in vivo human epidermis, consists of normal human keratinocytes cultured on an inert polycarbonate filter at the air–liquid interface in serum-free and chemically defined medium. The tissue cultures were maintained in the incubator at 37 °C, 5% CO_2_ and saturated humidity only for 48 h using SkinEthic Maintenance Medium (SMM). The medium was changed after 24 h and the treatments started after overnight incubation.

#### 4.8.2. Phototoxicity

To test the phototoxicity, the in vitro long UVA test, validated and approved by the EU Reference Laboratory for alternatives to animal testing (EURL ECVAM), was used (https://tsar.jrc.ec.europa.eu/test-method/tm1997-02, accessed on 4 March 2023). After the overnight incubation, tissues were transferred to fresh SMM (300 µL per well). Sterile pads were soaked with 20 µL of test samples, PTE (0.1% Fx) and 0.1% Fx solubilized in C12–C15 alkyl benzoate, and directly applied to the stratum corneum of the skin models [82]. Ketoprofen at 3% in C12–C15 alkyl benzoate, that is phototoxic, was used as a positive control [16], whereas C12–C15 alkyl benzoate was used as negative control. Eighteen hours after incubation of the test substances at 37 °C under a 95% air-5% CO_2_ atmosphere, the pads were removed, and the skin models were exposed to 6 J/cm^2^ UVA dose using the same instrument reported in Section 4.7. The same treatments were conducted in parallel on other non-irradiated tissues as a control. Three independent experiments in triplicate (*n* = 3) were carried out for each treatment.

##### Viability Assay

UVA-exposed and non-exposed tissues were rinsed 4 times with 0.5 mL phosphate-buffered saline (PBS) and incubated in 0.5 mg/mL MTT solution for 3 h at 37 °C, 5% CO_2_. After aspiration of the MTT medium and washing 4 times with 0.5 mL PBS, tissues were cut out of the inserts, and each-one transferred into glass tubes containing 1.25 mL isopropanol—40 mM HCl [82]. Formazan was extracted for 3 h under constant agitation in the dark. Three aliquots (200 µL) of each tube were pipetted in a 96-well plate and the viability of tissues was estimated by recording the absorbance at 540 nm with the microplate reader reported in the Section 4.6.2.

#### 4.8.3. Photoprotection

The photoprotective effect of PTE (0.1% Fx) and 0.1% Fx, both solubilized in C12–C15 alkyl benzoate, were tested on the RHE model by exposing the tissues to UVA (26 J/cm^2^) and UVB (1.5 J/cm^2^) doses that simulate the cumulative acute solar radiation (27.5 J/cm^2^) [81]. At this purpose, the same instrument reported in Section 4.7. was used. C12–C15 alkyl benzoate was used as negative control. Each tissue received 2 mg of test substances as recommended by guidelines on dermal absorption. After overnight incubation, a second application was performed 60 min before UVA and UVB irradiation. The same treatments were conducted in parallel on other non-irradiated tissues as control. Three independent experiments in triplicate (*n* = 3) were carried out for each treatment. At the end of experiments, culture media were harvested and stored immediately to −20 °C, whereas tissues were cut out of the inserts, and each-one transferred into Zero A—Formalin prefilled container (Histo-Line Laboratories, Milan, Italy) for histological analyses.

##### Lactate Dehydrogenase Assay

RHE models’ viability was evaluated by measuring the release of the cytosolic enzyme lactate dehydrogenase (LDH) in the culture medium by LDH activity assay kit (MAK066, Merck KGaA, Darmstadt, Germany), according to manufacturer’s instructions.

##### Reactive Oxygen Species (ROS) Assay

The intracellular ROS levels, expressed as percentage (%), were assessed according to Smeriglio et al. [83], by recording the fluorescence resulting from the intracellular oxidation of 2′,7′-dichlorofluorescin diacetate (DCF-DA, 10 µM in PBS), which was added to the culture medium 30 min before ending the tissue treatment. The medium was then removed, and the tissues, washed five times with PBS (pH 6.7), were lysed pipetting 500 μL of cold 0.1% Triton X-100. The resulting suspensions were diluted using PBS, and the fluorescence was recorded, by the same microplate reader reported in Section 4.6.1, at the following excitation and emission wavelengths: λ_ex_ 485 and λ_em_ 535, respectively.

##### Determination of IL-1α and Nitric Oxide (NO)

Interleukin-1α (IL-1α) release was measured by high-sensitivity human ELISA kit (RAB0269, Merck KGaA, Darmstadt, Germany) according to the manufacturer’s instructions, whereas NO release was evaluated by Griess reagent, according to Smeriglio et al. [83], by using sodium nitrite as reference standard (0–15 μM).

##### Histological Analysis

Tissues were formalin fixed (10% neutral buffered formalin) for 48 h, cut in the central part to have two equal halves, and then automatically processed with ASP6025S Leica Biosystems processor (Leica Biosystems, Wetzlar, Germany) as per manufacturer’s instructions. Samples were manually embedded, orienting the samples perpendicularly to the cut surface. From each formalin fixed paraffin embedded block, ten 4-µm-thick sequential sections were cut and stained with haematoxylin/eosin (H&E-6 sections) and immunohistochemistry (IHC—2 sections for each stain). IHC was performed using the BenchMark Ultra (Ventana Medical Systems, Roche Diagnostics Division, Hofman La Roche Ltd., Basel, Switzerland) automated immunostainer and visualization of the antibody–antigen reaction was via the indirect biotin-free method, Ultraview universal diaminobenzidine (DAB) detection kit (Ventana Medical Systems, Roche Diagnostics Division, Hofman La Roche Ltd., Basel, Switzerland). The following antibodies were used: Ki67 (MIB-1 clone, Dako, 1:100 dilution; 30 min heat pre-treatment, 32 min incubation time), and Cytokeratin 5&6 (D5/16B4 clone, Ventana, 1:1 dilution, 30 min heat pre-treatment, 32 min incubation time).

Each case was evaluated by two expert pathologists, blind to treatment. Evaluation was performed using a Leica DM 2000LED optical microscope (Leica Microsystems Inc, Wetzlar, Germany) and microphotographs were captured using the Leica Microsystems Flexacam C1 (Full-HD 1920 × 1080 pixels; Leica Microsystems Inc, Wetzlar, Germany) and Leica Application Suite X (LAS X) acquisition software (Leica Microsystems Inc, Wetzlar, Germany). Images were captured at 20× magnification, in the central part of the sample, avoiding the edge. Each image corresponds to a linear extension of tissue measuring 500 µm. Apoptotic/sunburn cells were counted in 5 different representative fields/sections of the H&E-stained slides, while Ki67 positive proliferating cells were evaluated counting positive nuclei in 3 different representative fields. Data were finally expressed as total number of all evaluations together and as mean of the different evaluations. Cytokeratin 5&6 was evaluated semi-quantitatively (mild, moderate, high expression), considering that apoptotic/sunburn cells show a more pronounced brown staining (as a possible effect of damage on cells cytoskeleton) compared to the surrounding cells.

### 4.9. Statistical Analysis

Three independent experiments in triplicate (*n* = 3) were carried out for each chemical analysis and in vitro cell-free and cell-based assay. The statistical significance was evaluated by one-way analysis of variance (ANOVA) followed by Student–Newman–Keuls Method using SigmaPlot 12.0 software (Systat Software Inc., San Jose, CA, USA).

Descriptive statistics including median and range of apoptotic/sunburn cells and Ki67-positive cells per case, was possible using the database. Differences in number of apoptotic/sunburn cells and Ki67 positive cells between different samples were calculated using T test calculator for three independent samples. Data were considered statistically significant for *p* < 0.05.

## 5. Conclusions

This study demonstrates that the culture conditions used increase the yield of algal biomass and favour the fusiformis morphotype, as also shown by the microscopic investigations. This morphotype shifts the algal metabolic pathway towards a higher production of carotenoids, as demonstrated by fluorescence microscopy. The extraction process used allowed to obtain an ethanolic extract very rich in carotenoids, notably xanthophylls, as demonstrated by phytochemical analyses. Fucoxanthin was the predominant compound, accounting for approximately 98% of the light-harvesting pigments. The titration of the whole extract, which showed a fucoxanthin content of about 7%, made it possible to evaluate the biological activity of the plant-complex with respect to that of pure fucoxanthin. Both fucoxanthin and the extract showed a marked antioxidant activity, excellent photostability, and an excellent safety profile, not inducing any phototoxic effect. In addition, an excellent skin photoprotection of the extract was observed, significantly reducing both the cytotoxicity and the release of oxidative and inflammatory stress markers on in vitro reconstructed human epidermis 3D model.

The complex of data shows that the ethanolic extract of *P. tricornutum*, even more than pure fucoxanthin, represents a promising skin protective plant-complex. These results could therefore be easily translatable in vivo for the development of broad-spectrum sun protection products.

## Figures and Tables

**Table 1:** Phytochemical profile of *Phaeodactylum tricornutum* ethanolic extract (PTE) elucidated by HPLC-DAD-APCI-MS/MS analysis. Results, which represent the mean ± standard deviation of three independent analyses in triplicate (*n* = 3), were expressed as mean peak area percentage (%), based on the diode array detection chromatograms acquired at 450 nm, with respect to the total area of identified compounds.

#	Compound	RT (min)	λ_max _ (nm)	[M+H]^+^ (*m*/*z*)	MS/MS (*m*/*z*)	Peak Area (%)
1	Chlorophyllide a	2.67	424, 665	614	596, 359, 312, 285	0.11 ± 0.01
2	E-Fucoxanthin	4.35	267, 448	642 ^a,b^	624, 582, 550, 489	77.45 ± 0.55
3	Chlorophyll c1	4.89	440, 637	612	494, 475	0.41 ± 0.02
4	(9Z)-Fucoxanthin	5.15	332, 438	660	642, 624, 581, 550	15.44 ± 0.24
5	(13Z)-Fucoxanthin	5.53	332, 438	660	642, 624, 581, 550	5.18 ± 0.03
6	(E)-Diadinoxanthin	6.13	450, 478	584	565, 221	0.47 ± 0.01
7	(E)-Diatoxanthin	7.75	450, 478	568	550, 515, 359, 305	0.45 ± 0.02
8	Chlorophyll a	8.37	430, 665	894	616, 844, 826, 592 542	0.28 ± 0.01
9	β-Carotene	13.32	458, 482	537 ^a^	413, 445	0.22 ± 0.01
	Chlorophylls					0.80
	Carotenoids					99.20
	Xanthophylls					98.99
	of which fucoxanthin					98.07
	Carotenes					0.22

^a^ Identified with commercially available standard; ^b^ [M + H-H_2_O]^+^.

## Data Availability

All data relevant to this study are contained within the article.

## Supplementary Materials

The following supporting information can be downloaded at: https://www.mdpi.com/article/10.3390/molecules28104190/s1, Figure S1, Chromatogram; Table S1, Commercially available photoprotective products derived from microalgae.

## Sample Availability

Samples of *Phaeodactylum tricornutum* ethanolic extract are available on request at info@archimedericerche.com.
