# Supplementary material for: Xanthophyll-Rich Extract of Phaeodactylum tricornutum Bohlin as New Photoprotective Cosmeceutical Agent: Safety and Efficacy Assessment on In Vitro Reconstructed Human Epidermis Model
Source: Molecules. 2023 May 19;28(10):4190. doi: 10.3390/molecules28104190 (PMC10222380; doi:10.3390/molecules28104190)
Supplement: Supplementary file 1 [file molecules-28-04190-s001.zip › molecules-2356555-supplementary.pdf]

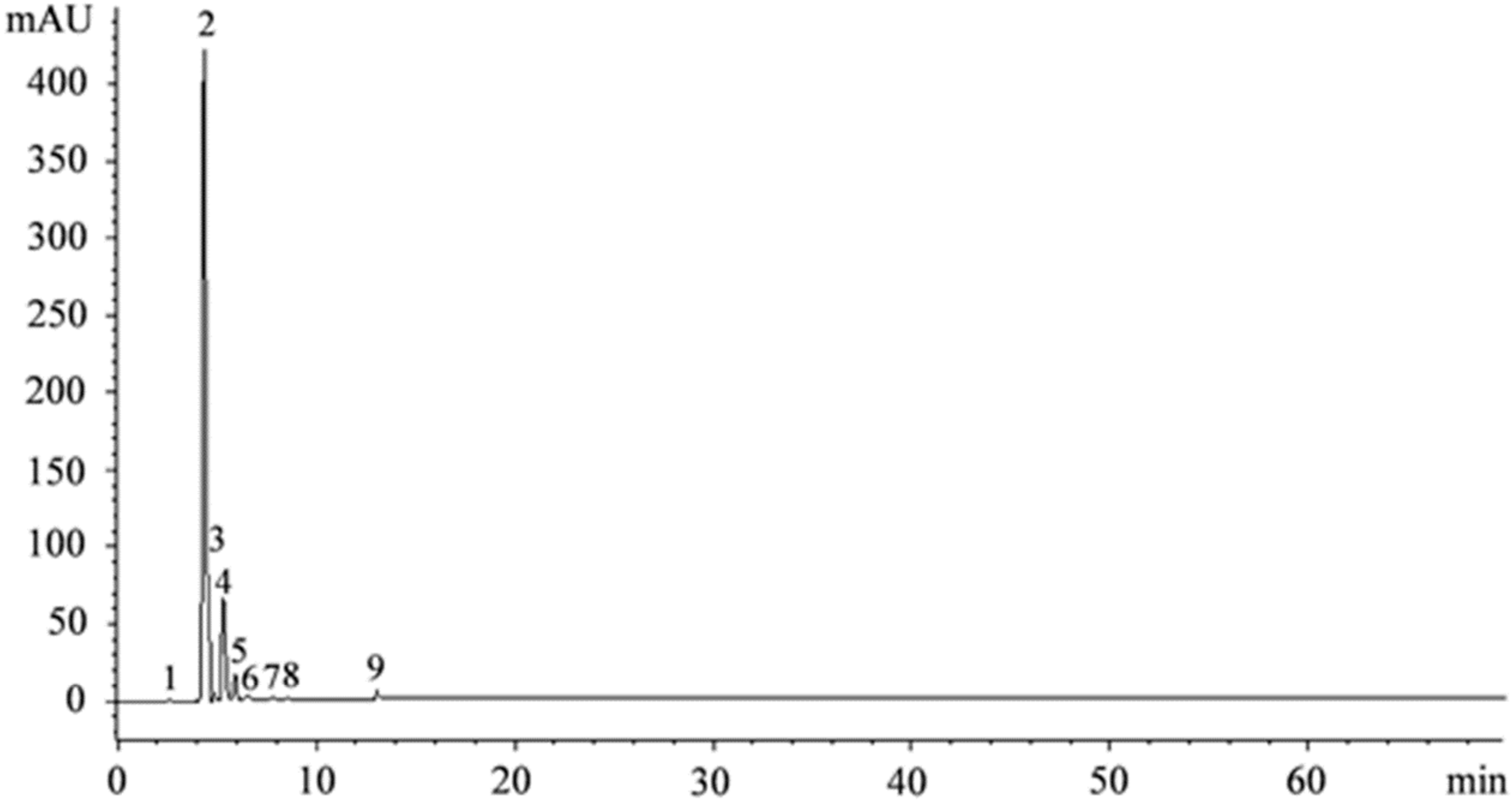

Figure S1. HPLC-DAD chromatogram of *Phaeodactylum tricornutum* ethanolic extract (PTE) acquired at 450 nm. Peak numbers refer to the elution order displayed in Table 1.

Table S1. Commercially available photoprotective products derived from microalgae.

Product Name	Source algae	Major constituents[a]	Biological activities
AstaDaily® (Dietary ingredient)	*Haematococcus pluvialis*	Astaxanthin Carotenoids	Antioxidant and anti-inflammatory properties
Phytobioactive Epsi-line® (Cosmetic)	Porphyridium. cru-entum	Polysaccharides	tanning and antioxi-dant properties
Renouvellance® (Cosmetic)	Porphyridium. cru-entum	Phycobilins Polysaccharides	Photo protection, moisturizing
Sublime Defense® (Cosmetic)	*Parachlorella beijerinckii*	exopolysaccharides	Broad-spectrum UVA/UVB protec-tion
SunChlorella Cream® (Cosmetic)	*Chlorella vulgaris*	Chlorophyll Carotenoids Vitamin E	Increased skin hy-dration and elastic-ity
Sunsafe Rx® (Dietary Ingredient)	*Haematococcus pluvialis*	Astaxanthin Lutein Zeaxanthin Lycopene Omega 3	Improved skin elas-ticity, reduced fine lines and wrinkles

[a] Compounds listed in INCI order.
